# Electrochemiluminescence at 3D Printed Titanium Electrodes

**DOI:** 10.3389/fchem.2021.662810

**Published:** 2021-05-25

**Authors:** Samantha F. Douman, Miren Ruiz De Eguilaz, Loanda R. Cumba, Stephen Beirne, Gordon G. Wallace, Zhilian Yue, Emmanuel I. Iwuoha, Robert J. Forster

**Affiliations:** ^1^National Centre for Sensor Research, Chemistry Department, Dublin City University, Dublin, Ireland; ^2^SensorLab (University of the Western Cape Sensor Laboratories), University of Western Cape, Cape Town, South Africa; ^3^Australian Research Council, Centre of Excellence for Electromaterials Science, Intelligent Polymer Research Institute, University of Wollongong, Wollongong, NSW, Australia; ^4^FutureNeuro SFI Research Centre, Dublin, Ireland

**Keywords:** electrochemiluminescence, 3D-electrode array, voltammetry, annihilation and co-reactant system, heterogeneous electron transfer kinetics

## Abstract

The fabrication and electrochemical properties of a 3D printed titanium electrode array are described. The array comprises 25 round cylinders (0.015 cm radius, 0.3 cm high) that are evenly separated on a 0.48 × 0.48 cm square porous base (total geometric area of 1.32 cm^2^). The electrochemically active surface area consists of fused titanium particles and exhibits a large roughness factor ≈17. In acidic, oxygenated solution, the available potential window is from ~-0.3 to +1.2 V. The voltammetric response of ferrocyanide is quasi-reversible arising from slow heterogeneous electron transfer due to the presence of a native/oxidatively formed oxide. Unlike other metal electrodes, both [Ru(bpy)_3_]^1+^ and [Ru(bpy)_3_]^3+^ can be created in *aqueous* solutions which enables electrochemiluminescence to be generated by an annihilation mechanism. Depositing a thin gold layer significantly increases the standard heterogeneous electron transfer rate constant, k^o^, by a factor of ~80 to a value of 8.0 ± 0.4 × 10^−3^ cm s^−1^ and the voltammetry of ferrocyanide becomes reversible. The titanium and gold coated arrays generate electrochemiluminescence using tri-propyl amine as a co-reactant. However, the intensity of the gold-coated array is between 30 (high scan rate) and 100-fold (slow scan rates) higher at the gold coated arrays. Moreover, while the voltammetry of the luminophore is dominated by semi-infinite linear diffusion, the ECL response is significantly influenced by radial diffusion to the individual microcylinders of the array.

## Introduction

Three-dimensional (3D) electrodes offer significant advantages in areas such as highly sensitive electrochemical analysis, electronic devices (Wang et al., 2015), and energy storage devices (Ambrosi and Pumera, 2016). In particular, 3D electrodes can be highly beneficial for understanding the electrochemical properties of biological systems where the organisation of components in 3D directly affects their function and transport properties (Heuschkel et al., 2002; Durmus et al., 2013; Xie et al., 2015). Moreover, arrays of cylindrical electrodes can have enhanced properties due to unique combinations of radial transport to the electrode tips while linear diffusion dominates transport to the side walls. Thus, higher Faradaic currents and even higher signal-to-noise ratios may be observed (Minter, 2013). Furthermore, generator-collector type experiments may be possible in 3D due to the short diffusion distance (Chabi et al., 2013). 3D architectures with length scales of hundreds of microns can be conveniently fabricated *via* 3D printing or additive manufacturing (AM) (Ambrosi et al., 2015; Xu et al., 2017; Sharafeldin et al., 2018). While significant effort has been invested in elucidating the electrochemical properties of these 3D arrays (Ambrosi and Pumera, 2016), their ability to generate light through electrochemiluminescence, ECL, has been less widely considered (Gao et al., 2017; Lv et al., 2020; Ma et al., 2020). This is an important objective since both the redox and excited state dynamics, as well as, mass transport, e.g., of the luminophore and co-reactant to the electrode surface, collision of the activated form of the luminophore and co-reactant in solution, influence the intensity of the light generated and these parameters are influenced by the structure and geometry of the 3D array and the surface composition. For example, an oxide layer forms spontaneously on some metals that can be 3D printed, e.g., titanium, which opens up the possibility of inhibiting the water reduction reaction allowing the reduced form of key ECL luminophores, e.g., those based on [Ru(bpy)_3_]^2+^, to be electrogenerated in aqueous solutions.

In this contribution, we report on the electrochemical and electrochemiluminescence, ECL, properties of a 3D printed titanium microcylinder array working electrode. Selective Laser Melting (SLM) (Yap et al., 2016; Huang et al., 2017; Xiao et al., 2019), has been used to produce 3D Ti_6_Al_4_V electrodes. This additive manufacturing approach uses a high-power laser to incrementally melt and fuse thin layers of metal powder to create a custom electrode according to a pre-designed CAD file (Zhao et al., 2014; Xuetong et al., 2019). Compared to lithography, SLM provides a facile and cost-effective approach to produce both micro and macro structures. Here, microscopy, HR-SEM, cyclic voltammetry and electrochemiluminescence have been used to characterise the 3D electrodes, especially the surface roughness and the impact of the electrode geometry and composition on the electrochemical properties. Significantly, unlike traditional metal electrodes, we show that [Ru(bpy)_3_]^1+^ can be electrogenerated allowing ECL to be generated by an annihilation mechanism avoiding the need for a co-reactant. Moreover, co-reactant ECL can be generated from [Ru(bpy)_3_]^2+^ using tri-propyl amine, TPA, as a co-reactant.

We also demonstrate the ability to electrodeposit a thin, conformal, gold layer onto the electrodes without significant “shadowing” by adjacent electrodes. This approach is attractive since a “scaffold” can be 3D printed and then modified to optimise the properties for particular applications. For example, coating the titanium array with gold increases the rate of heterogeneous electron transfer converting the quasi-reversible response observed at the native 3D titanium electrode into a fully reversible response. This increase in the rate of heterogeneous electron transfer causes a significant increase in the intensity of the ECL and causes radial diffusion to exert a greater influence over the co-reactant ECL. The combination of high-resolution 3D printing followed by electrodeposition of a different metal opens the possibility of creating 3D structures with interesting properties, e.g., plasmonically enhanced ECL, where both the electrode structure and composition influence the voltammetric response.

## Experimental Methods

### Reagents and Materials

Potassium ferro/ferricyanide (Fe(CN)_6_^3−/4−^), Tris(2,2′- bipyridyl)dichlororuthenium(II) hexahydrate (Ru(bpy)_3_Cl_2_·6H_2_O), tripropylamine (TPA), sulfuric acid (H_2_SO_4_), and phosphate buffered solution (PBS, pH 7.4) were all purchased from Sigma Aldrich and used as received. Aqueous gold plating solution (Technic-Gold 25 ES RTU) was purchased from Technic Ink, UK. All solutions were prepared using Milli-Q water (18 MΩ cm).

### Instrumentation

Electrochemical experiments were performed using a CH Instrument, Model 760B Potentiostat. The electrochemical experiments were configured in a three-electrode setup where the three-dimensional (3D) electrode was the working electrode, with a BAS silver/silver chloride (Ag/AgCl) as the reference electrode and a platinum wire (Sigma Aldrich) as the counter electrode. Electrogenerated chemiluminescence (ECL) measurements were performed with an Oriel 70680 photomultiplier tube (PMT) biassed at −850 V using a high-voltage power supply (Oriel, Model 70705) and an amplifier/recorder (Oriel, Model 70701). Scanning electron microscopy (SEM) and energy dispersive X-ray spectroscopy (EDX) was performed using a Hitachi S5500 Field Emission SEM.

### Fabrication of 3D-Printed Electrode

3D printed titanium (Ti) electrodes were fabricated according to a procedure previously reported by Zhao et al. (2014). In brief, the electrode design was drawn using SolidWorks modelling software. Metal 3D printing was carried out with a Realizer SLM50 metal printer (Realizer, Germany) using the Selective Laser Melting (SLM) technique. A focused, high-energy laser beam fused and linked Ti alloy (Ti-6AI-4V) powder on a printing stage in a layer by layer fashion to create an array of 25 vertical round microcylinders (0.015 cm radius, 0.3 cm high) spaced evenly on a 0.48 × 0.48 cm square base that contained 0.75 × 0.75 mm holes.

### Surface Modification

The fabricated 3D printed electrodes were cleaned by sonication in a 50:50 v/v milli-Q water/ethanol solution before use. The electrodes were then dried under a stream of nitrogen. The aqueous gold plating solution was first deoxygenated with nitrogen for 30 min prior to deposition. The 3D printed electrodes were then immersed into the aqueous gold plating solution in the presence of an Ag/AgCl reference electrode and a platinum counter electrode. Gold was potentiostatically deposited at −0.9 V while measuring the charge passed. The deposition time used was 1000 s. Following electrodeposition, the 3D electrodes were electrochemically cleaned by cycling in an aqueous solution containing 0.1 M H_2_SO_4_ as supporting electrolyte. The 3D electrodes were then rinsed thoroughly with milli-Q water before use.

## Results and Discussion

### Structural Characterisation

**Figure 1:**
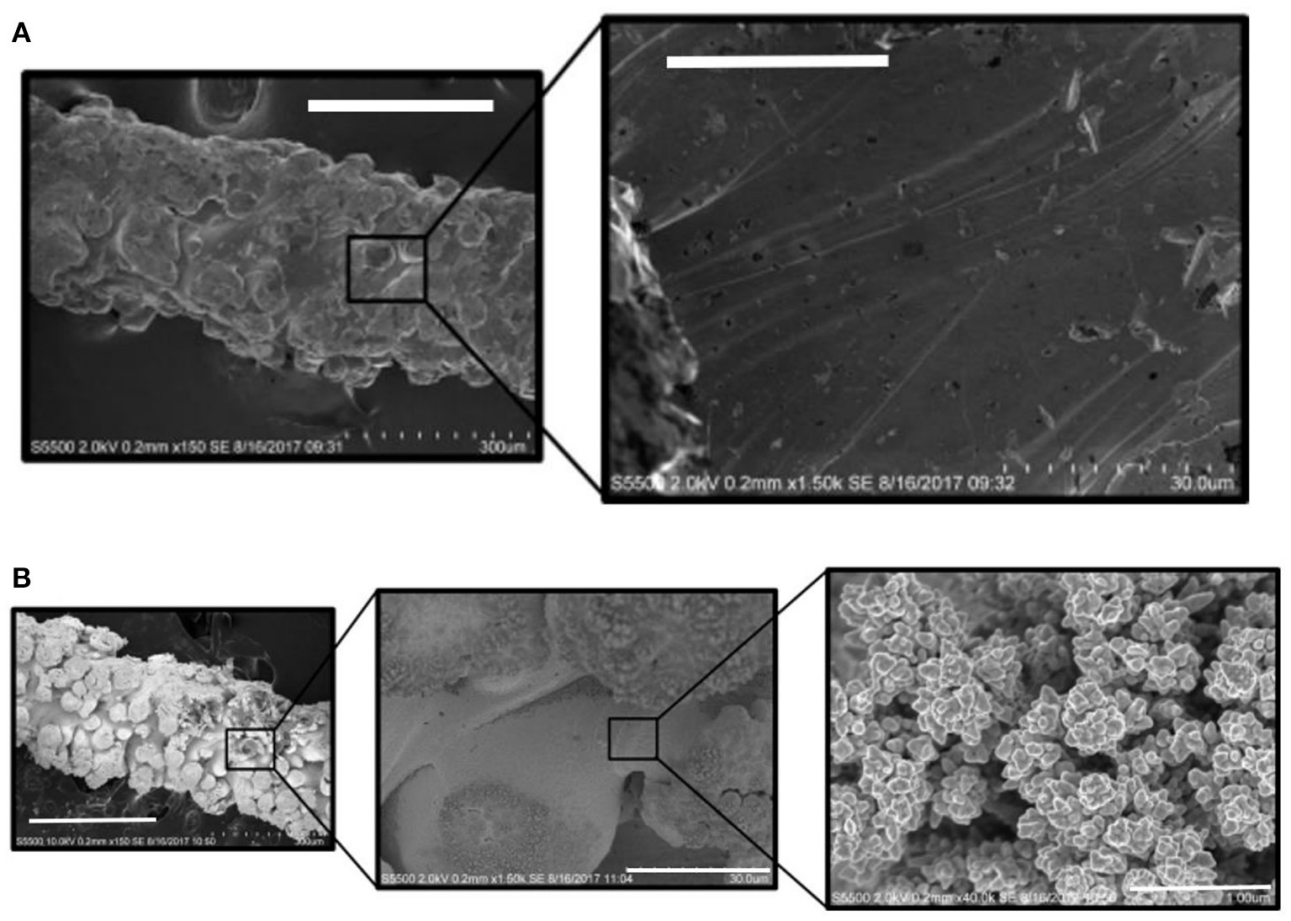
Secondary electrons HR-SEM micrographs showing **(A)** 3D Ti and **(B)** Au-coated 3D Ti electrodes, at low and high magnifications, using accelerating voltages that range from 2 to 10 kV, respectively. The scale bars are: **(A)** Left to right: 300 and 30 μm. **(B)** Left to right: 300, 30 and 1 μm.

The electrochemical performance of the 3D printed structure depends on the spatial distribution of the electrode features (microcylinders) as well as their diameter and separation. The performance is further influenced by the surface composition (oxide layer formation) and surface roughness. HR-SEM micrographs of one of the electrode microcylinders reveals that the SLM fabrication process produces a rough surface consisting of fused titanium grains (Figure 1A; Supplementary Figure 1). The EDX spectrum (Supplementary Figure 2) is dominated by peaks associated with Ti, but carbon peaks are also present due to adventitious impurities, as well as aluminium and vanadium which are minor components of the SLM powder.

### Electrochemical Properties

Electrochemiluminescence can be generated from [Ru(bpy)_3_]^2+^ systems (bpy = 2,2′-bipyridyl) through different mechanisms including “annihilation” between the [Ru(bpy)_3_]^+^ and [Ru(bpy)_3_]^3+^ ions or reaction of the reduced or oxidised forms with an electrogenerated co-reactant, such as peroxydisulphate or tri-propyl amine (Li et al., 2017). In contrast to the co-reactant pathway, annihilation is generally not possible in *aqueous* solutions at conventional metal electrodes, e.g., platinum, since the reduction potential is significantly more negative than the hydrogen evolution reaction. However, Bard and co-workers demonstrated that “hot” electrons can be generated in *aqueous* solution at oxide covered tantalum electrodes and used to generate [Ru(bpy)_3_]^+^ in an aqueous solution (Gaillard et al., 1999). In the presence of [Ru(bpy)_3_]^3+^, the two ions react to form the electronically excited state, [Ru(bpy)_3_]^2+*^, that can then electrochemiluminesce at ~610 nm. A key objective is to understand the feasibility of generating the reduced ruthenium tris-bpy ion using a 3D printed titanium electrode and generating ECL.

**Figure 2:**
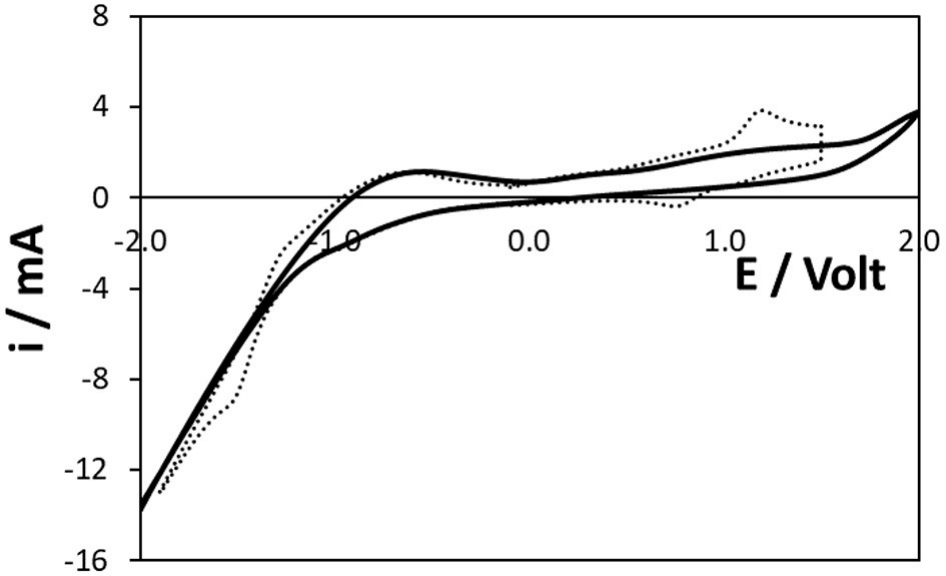
Voltammograms of the 3D Ti electrode in pure electrolyte (solid line) and a 10 mM solution of [Ru(bpy)_3_]^2+^ (dashed line). The electrode had a native oxide layer and the quiet time at 0.000 V was 5 s. The supporting electrolyte is aqueous 0.1 M H_2_SO_4_. The scan rate is 0.1 V s^−1^. The solution contains oxygen.

Cyclic voltammetry was used to determine the available potential window and to gain an insight into the electrochemically active area of the 3D Ti electrodes and hence their surface roughness. Figure 2 shows the voltammogram obtained in 0.1 M H_2_SO_4_ as supporting electrolyte in the range −2.0 to +2.0 V at a scan rate of 0.1 V s^−1^. As described by Burrell and Armstrong (1986), exposure to air or application of potentials more positive than ~+0.2 to +0.3 V results in the formation of a TiO_2_ layer. It should be noted that Ti oxide films form spontaneously and rapidly on the surface of pure Ti (Li et al., 2011). For Ti alloys, thicker oxide layers can form and can reach 10–20 μm thick (Vasilescu et al., 2017). Figure 2 is consistent with the behaviour observed for pure titanium electrodes not produced using SLM. The onset potential for oxide formation, ~+0.15 V, is similar for both 3D printed and pristine titanium disk electrodes, suggesting that the TiO_2_ layer on the 3D electrodes is comparable to that formed on pristine titanium. Moreover, the close to constant oxidation current observed between ~0.0 and +1.6 V suggests that the oxide layer grows under conditions of constant internal electric field and its thickness depends on the potentials applied and the electrolysis time. The impact of the oxide layer on the dynamics of [Ru(bpy)_3_]^2+^ oxidation, essential for ECL generation by either the co-reactant or annihilation pathways, is considered in a later section.

Significantly, hydrogen ion reduction is only observed for potentials more negative than ~-1.2 V and even at the potential where one might expect reduction of [Ru(bpy)_3_]^2+^, −1.5 V, the reduction is very inefficient compared to conventional electrodes. For example, the current density at the titanium array at −1.000 V and a scan rate of 0.1 V s^−1^ is 1.7 ± 0.2 mA cm^−2^, whereas at carbon, gold and platinum, the values are 7.1 ± 0.9 mA cm^−2^, 15.0 ± 2.2 mA cm^−2^ and >200 mA cm^−2^, respectively.

This result suggests that it may be possible to generate ECL through the annihilation pathway in *aqueous* solutions using these 3D printed electrode arrays.

**Figure 3:**
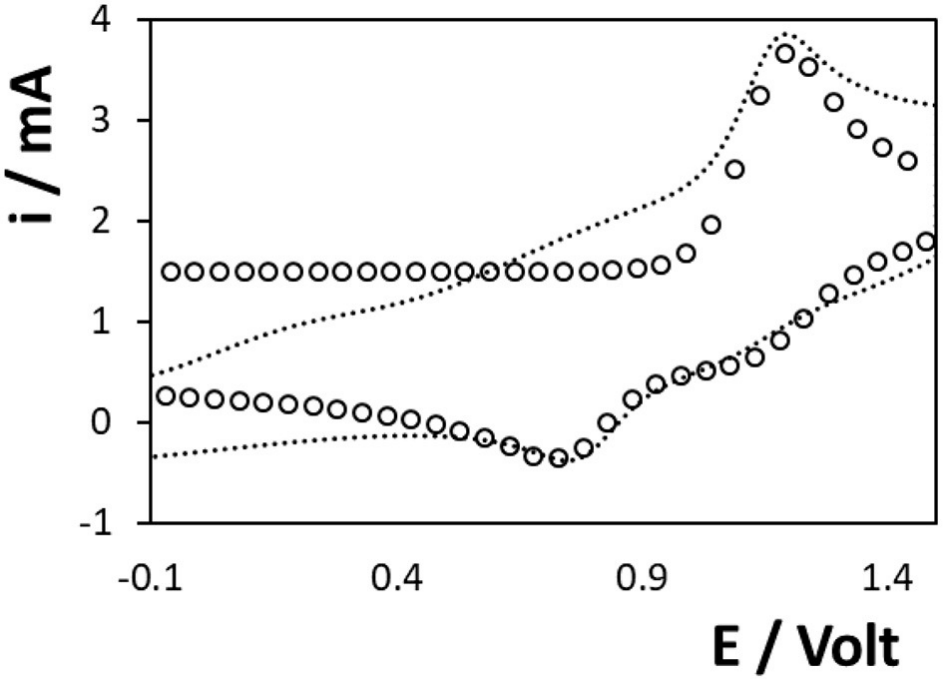
Voltammogram of a 10 mM solution of [Ru(bpy)_3_]^2+^ at the 3D Ti electrode. Before recording the CV, the electrode was preconditioned at −1.0 V, where the oxide is reduced, for 60 s followed immediately by the voltammetric scan with no quiet time. The supporting electrolyte is aqueous 0.1 M H_2_SO_4_. The scan rate is 0.1 V s^−1^. The open circles represent the best fit simulated voltammogram in which D is 1.1 × 10^−5^ cm^2^ s^−1^ and k^o^ is 1.1 × 10^−4^ cm s^−1^.

The electrogeneration of the anion and cation radicals depends on heterogeneous electron transfer to/from the Ru^2+^ complex. The dashed line of Figures 2, 3 show the cyclic voltammogram for an aqueous 10 mM solution of [Ru(bpy)_3_]^2+^ at the 3D titanium electrode array. Despite holding the potential at −1.0 V for 60 s in Figure 3 in order to reduce the surface oxide, the currents, peak potentials and peak-to-peak separations, ΔE_P_, observed in Figures 2, 3 are indistinguishable. This result suggests that an oxide layer forms when the potential is scanned from −0.1 V to the formal potential of the Ru^2+/3+^ couple, ~+0.965 V. Moreover, the similarity of the ΔE_P_ values suggests that the rates of heterogeneous electron transfer are similar with and without the preconditioning step at −1.0 V. This result is consistent with the thickness of the oxide layer being dominated by cycling at positive potentials rather than the initial coverage of the oxide. As reported previously for related systems (Zhang, 2020), deoxygenating the solution decreases the rate at which the oxide layer forms. In deoxygenated solution the Ru^2+/3+^ voltammetry appears more reversible, i.e., ΔE_P_ is 210 ± 45 mV for the first five full cycles between −2.000 and +1.500 V. However, ΔE_P_ increases as the number of scans increases and the response obtained after 15–20 scans is indistinguishable from that shown in Figure 2. This result suggests that oxide forms at positive potentials even in the absence of dissolved oxygen. Moreover, it is important to note that exposure to air and ageing of the oxide, e.g., over several months, causes the magnitude of the currents associated with the Ru^2+/3+^ couple shown in Figure 2 to decrease by as much as 75%. For example, the peak current associated with the Ru^2+/3+^ couple shown in Figure 2 decreases from 3.6 ± 0.2 to 0.8 ± 0.3 mA following storage in air for 6 months.

The observation of peak shaped rather than sigmoidal responses suggests that despite the diameter of the cylinder electrodes being of the order of 300 μm semi-infinite linear, rather than radial, diffusion dominates the response at this scan rate. Semi-infinite linear diffusion would be expected if the depletion zones around each cylinder electrode coalesced and the entire volume within the 3D array was electrolysed. However, based on a diffusion coefficient of 1.1 × 10^−5^ cm^2^ s^−1^, the depletion/diffusion layer thickness, δ, [≈(D t)^1/2^] is ~3.3 × 10^−4^ cm. This value is significantly smaller than the separation between the individual electrode microcylinders (0.1 cm) and the depletion zones surrounding each microcylinder do not overlap under these relatively short timescale conditions. For microcylinder electrodes, Aoki has derived equations that allow the relative contributions from radial diffusion to the tip of the microcylinder and linear diffusion to the main body of the electrode (Aoki, 1985) to be calculated. Given a radius of 0.015 cm and a microcylinder length of 0.3 cm, at 100 mV s^−1^, the current arising from linear diffusion is expected to be ~ 20 times larger than the contribution from radial diffusion to the electrode tip (Fang, 2009).

Even at the relatively slow scan of 0.1 V s^−1^, the peak-to-peak separation, ΔE_P_, is significantly larger than the 57 mV expected for an ideally reversible couple under semi-infinite linear diffusion control. This behaviour is consistent with slow heterogeneous electron transfer most likely due the presence of the surface oxide. Figure 3 shows the best fit to the experimental response in which the diffusion coefficient and formal potential are fixed at the values determined using a glassy carbon electrode, i.e., 1.1 × 10^−5^ cm^2^ s^−1^ and 0.965 V, respectively, and the standard heterogeneous electron transfer, k^o^, is the only freely adjustable parameter. While there is general agreement between the experimental response and the currents, the overall quality of the fit is not ideal. However, since our primary objective is to understand the influence of heterogeneous electron transfer dynamics, k^o^ is the only variable and effects, such as diffusion to the tips of the microcylinders, electrode roughness etc. are not included. Significantly, there is close agreement between the experimental and model peak potentials using a k^o^ of 1.1 × 10^−4^ cm s^−1^. This value is at least 1,000-fold slower than that observed at oxide free, pristine electrodes which reflects the presence of an oxide coating on the electrode surface which significantly slows electron transfer.

Figure 2 also shows the reduction of [Ru(bpy)_3_]^2+^ with a formal potential of ~-1.43 V. While recognising that the significant background current due to water electrolysis makes it challenging to accurately determine peak potentials, the ΔE_P_ is significantly smaller for the Ru^2+/1+^ (ΔE_P_ = 0.25 V) couple than that observed for Ru^2+/3+^ (ΔE_P_ = 0.44 V). This result suggests that the rate of heterogeneous electron transfer is significantly larger for reduction than oxidation. While accurate correction for the background limits the accuracy, simulations using a semi-infinite linear diffusion model suggests that k^o^′(Ru^2+/1+^) is ~2 × 10^−3^ cm s^−1^, i.e., ~20-fold faster than Ru^2+/3+^. The intrinsic properties of the complex are likely to influence this behaviour, e.g., differences in the solvent reorganisation energy, but it is perhaps important to note that the reduction proceeds at a potential where the oxide layer is either not present or significantly thinner. Overall, these results demonstrate that the titanium electrode array is capable of both oxidising and reducing [Ru(bpy)_3_]^2+^ in *aqueous* electrolyte. This opens up the possibility of generating ECL by an annihilation mechanism thus avoiding the need to add a co-reactant.

### Annihilation Electrochemiluminescence

3D electrodes open up the possibility of electrochemically generating light throughout a 3D volume rather than at a 2D planar surface. Moreover, depending on the geometry of the 3D electrode, it may be possible to selectively enhance the brightness of the emission in particular regions, e.g., due to enhanced rates of mass transport arising from radial diffusion to the top of the individual microcylinder electrodes.

Double potential step chronoamperometry represents a useful approach to creating the reduced and oxidised forms of ruthenium tris bipyridyl required for ECL generation by annihilation. In this approach, the oxidised species diffusing out from the electrode during the forward pulse will diffuse towards the electrode during the reverse pulse and meet the electrogenerated reduced species in a reaction zone adjacent to the electrode. These two forms then undergo a highly exergonic reaction to create the excited state [Ru(bpy)_3_]^2+*^, that relaxes to the ground state by emission at 610 nm.

**Figure 4:**
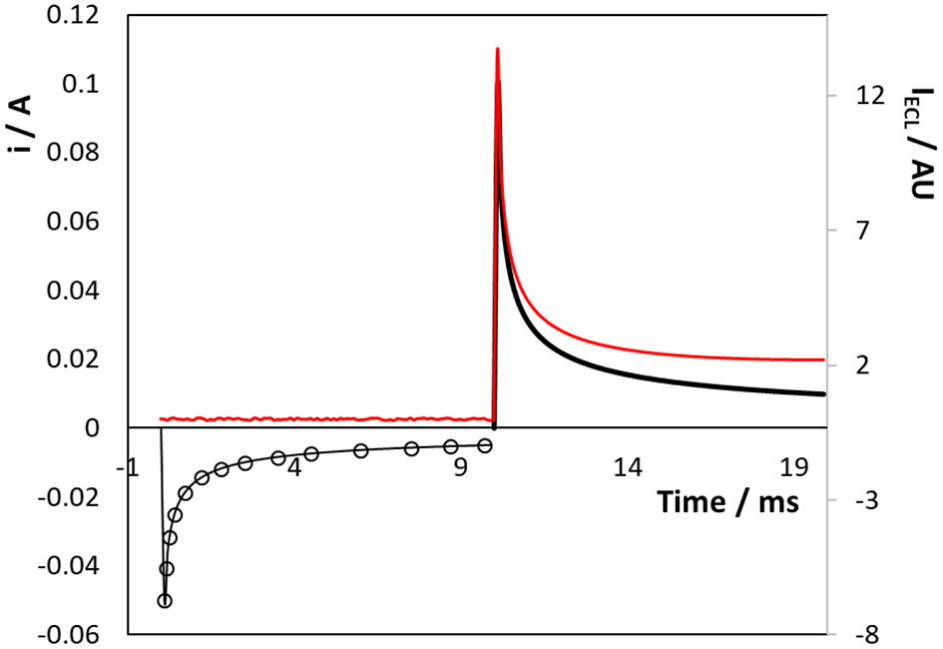
Transient current (black line) and ECL (red line) responses for the 3D titanium array coated with a native oxide following potential steps from 0.000 to +1.200 V (0–10 ms) and then to −1.600 V (10–20 ms) vs. Ag/AgCl. Reduction currents are positive. The supporting electrolyte is aqueous 0. 1 M H_2_SO_4_.

Figure 4 shows the current and ECL response when a titanium electrode array that is coated with a native oxide layer is subjected to a double potential step in the presence of 50 μM [Ru(bpy)_3_]^2+^ dissolved in aqueous in 0.1 M H_2_SO_4_ as supporting electrolyte. The Ru^3+^ species is first generated by stepping to a potential of +1.200 V, i.e., sufficiently positive of the Ru^2+/3+^ formal potential to generate Ru^3+^ at a diffusion-controlled rate for 10 ms. Then, [Ru(bpy)_3_]^1+^ was electrogenerated by stepping the potential to −1.600 V, again for 10 ms. The current response for the first step (Ru^2+^ oxidation) can be accurately modelled (open circles Figure 4) using the Cottrell equation and a diffusion coefficient of 1.1 × 10^−5^ cm^2^ s^−1^. Significantly, despite the surface roughness observed in both the SEM images, the forward step chronoamperometry data is best fitted using an electrode area of 2.9 cm^2^, i.e., a surface roughness factor of ~2.2. The issue of surface roughness is revisited in a later section dealing with the formation of an oxide monolayer on a conformal gold layer electrodeposited on the Ti array. The adequacy of the fitting based on the Cottrell equation suggests that even at 10 ms the depletion layer thickness is comparable to the length scale of a significant fraction of the surface roughness and the rough electrode appears relatively smooth to diffusing molecules. The magnitude of the current for the second step (Ru^2+^ reduction) is significantly larger than the first step and is not satisfactorily modelled using the Cottrell equation. These deviations reflect the fact that [Ru(bpy)_3_]^2+^ reduction and the hydrogen evolution reaction, HER, proceed in parallel when the potential is stepped to −1.600 V.

Figure 4 also shows the intensity of the light (red line) generated during the individual potential steps. Significantly, no ECL is observed until the second pulse is executed and both the oxidised and reduced forms are present in the diffusion layer. This result is important since it suggests that even in aqueous media, [Ru(bpy)_3_]^1+^ can be generated at negative potentials at oxide covered titanium electrodes. The ECL response decays less rapidly than the current associated with Ru^3+^ production suggesting that the nature of the diffusional mass transport may change, e.g., a contribution from radial mass transport, due to the small size of the microcylinders of the 3D array. The ability to generate ECL in an aqueous solution at a 3D electrode without the need for a co-reactant is potentially very useful for diverse applications including “reagentless” sensors and display devices.

**Figure 5:**
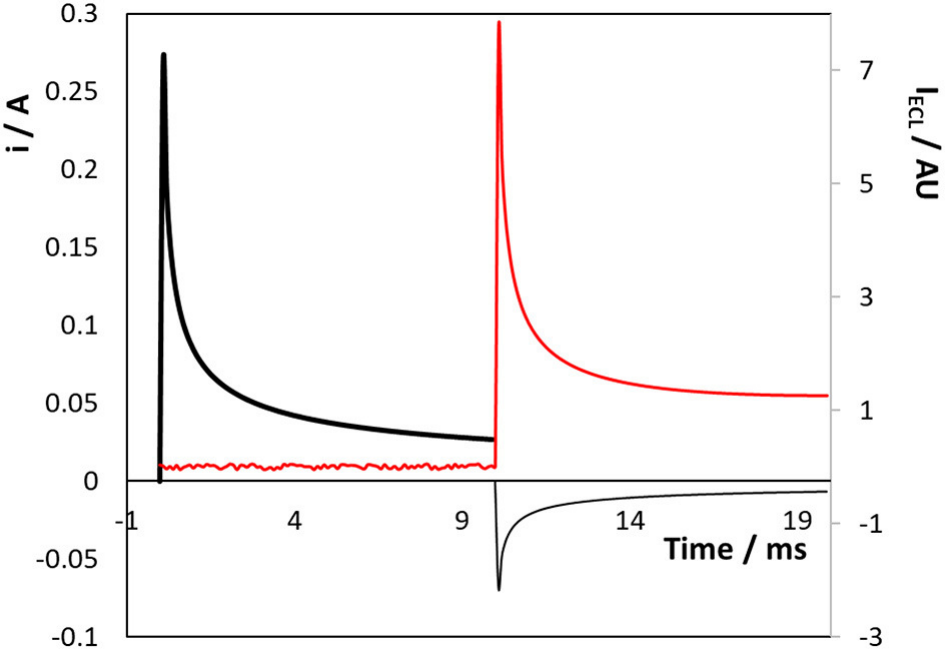
Transient current (black line) and ECL (red line) responses for the 3D titanium array coated with a native oxide following potential steps from 0.000 to −1.600 V (Ru^2+^ reduction, thick black line) and then to +1.200 V (Ru^2+^ oxidation, thin black line) vs. Ag/AgCl. Reduction currents are positive.

Figure 5 shows the effect of changing the direction of the potential step to first create [Ru(bpy)_3_]^1+^ followed by [Ru(bpy)_3_]^3+^. As before, no ECL is observed until the second potential step is applied, i.e., creating Ru^1+^ or Ru^3+^ alone does not lead to emission. While the current transients follow a similar behaviour as that shown in Figure 4, i.e., reduction being more complicated due to water electrolysis, the ECL intensity is significantly lower (~35%) than when the oxidation step is applied first. This result most likely arises because water electrolysis perturbs the depletion layer, e.g., localised stirring due to microbubble formation, as the Ru^2+^ reduction step proceeds.

These results demonstrate that, unlike traditional electrode materials such as gold or platinum, [Ru(bpy)_3_]^2+^ can be both oxidised and reduced in aqueous solutions using 3D titanium arrays formed using sintered laser melting. The ability to create both radical cations and anions allows electrochemiluminescence to be generated in *aqueous* solution using an annihilation mechanism.

### Co-reactant Electrochemiluminescence

The use of a co-reactant allows ECL to be generated from either Ru^3+^ or Ru^1+^ alone. Here, the focus is on ECL generation from the Ru^3+^ species using tri-propyl amine, TPA, as the co-reactant. As well as using the native arrays, the properties of arrays functionalised with an electrodeposited gold coating, were investigated.

#### Properties of Gold Coated Arrays

The 3D printed titanium array was coated with a thin, conformal gold layer using electrodeposition. For potentials less positive than ~+1.2 V, the gold surface will be oxide free which is expected to lead to a higher rate of heterogeneous electron transfer thus allowing the effect of the 3D structure of the array on diffusional transport to be more clearly observed. Also, an oxide monolayer can be formed and subsequently reduced by cycling in dilute acid. The charge passed during reduction of the gold oxide allows the microscopic surface area and hence the surface roughness to be accurately determined. This is challenging on the clean titanium since a well-defined oxide reduction peak is not observed. The charge associated with the reduction of gold oxide is ~390 μC cm^−2^. Thus, by integrating the area under the gold oxide reduction peak in the background corrected cyclic voltammograms recorded in acidic (0.1 M H_2_SO_4_) electrolyte it is possible to measure the microscopic surface area of the gold electrode (Burke, 1997). For the gold coated array, the charge passed, 9.2 ± 0.9 × 10^−3^ C, corresponds to a microscopic area of 23.5 ± 2.4 cm^2^. The geometric area of the array is 1.32 cm^2^, which, when taken in conjunction with the microscopic area, gives a significant surface roughness of 17.8 ± 1.8. The microscopic surface roughness is significantly larger than that found for polished gold electrodes where values are typically between 1.2 and 2.5 (Romanowski, 1977; Menshykau, 2008). This large value is consistent with the HR-SEM images shown in Figure 1 which indicates that the laser fusion 3D deposition method produces a rough, granular structure reflecting the particulate nature of the starting material. Significantly, HR-SEM indicates that the gold deposit exists as a thin (<300 nm) conformal layer over the array making it likely that the roughness of the underlying titanium electrode array is similar or possibly larger. It is important to note that the surface roughness determined by reducing the surface oxide (17.8 ± 1.8) is significantly larger than the value obtained by fitting the chronoamperometry response shown in Figure 4 (2.2). This result suggests that even at timescales of 10 ms, the depletion layer thickness (~3.5 μm) exceeds the length scale of a significant fraction of the surface roughness.

**Figure 6:**
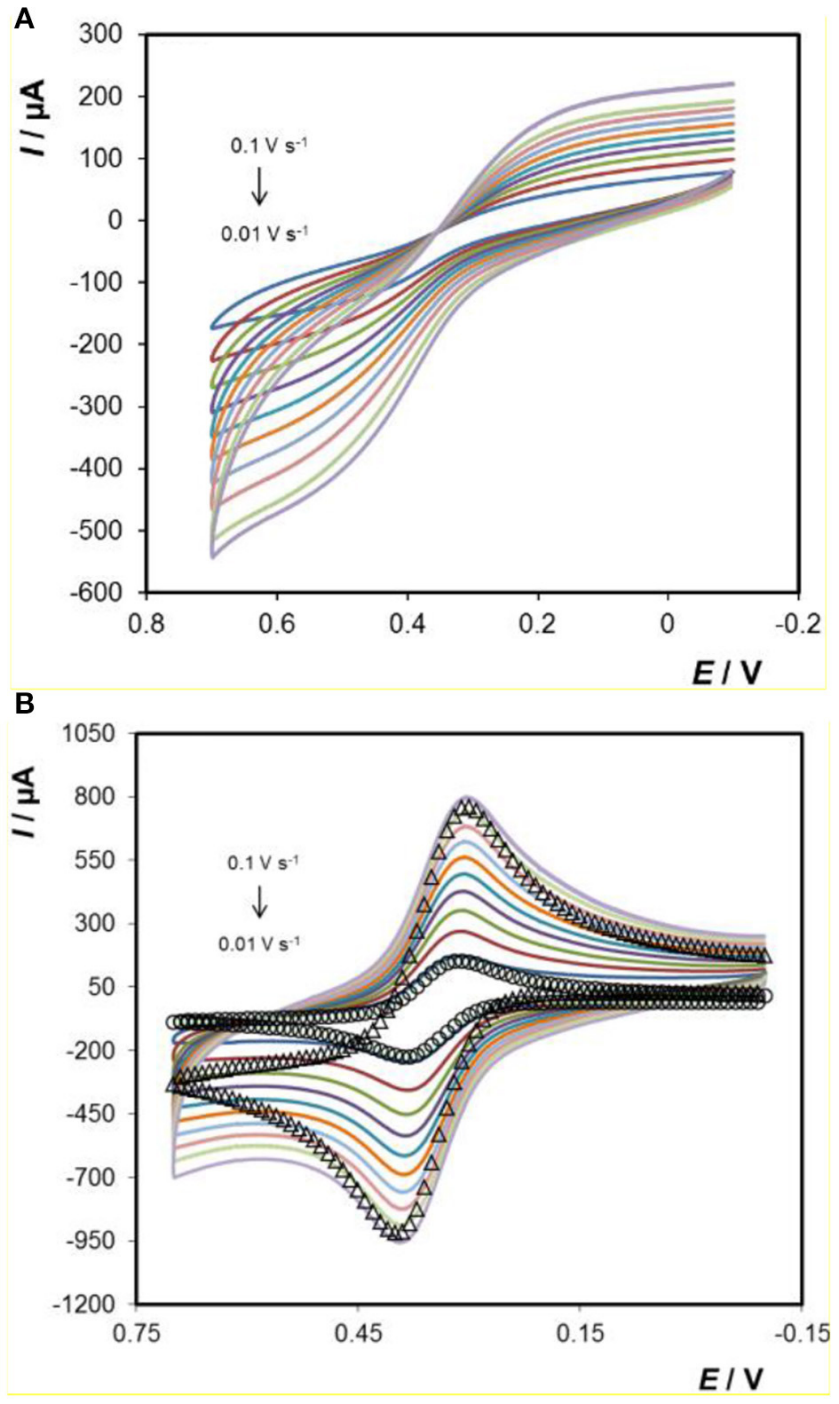
Voltammograms of **(A)** 3D Ti electrode and **(B)** Au-coated 3D Ti electrode in 1 mM ferrocyanide in 0.1 M H_2_SO_4_ as supporting electrolyte. From smallest to largest peak currents, the scan rates are 0.01, 0.02, 0.03, 0.04, 0.05, 0.06, 0.07, 0.08, 0.09, and 0.1 V s^−1^. The open circles (°) represent the best fit under semi-infinite linear diffusion control where the standard rate of heterogeneous electron transfer, k^o^, is 8 ± 0.4 × 10^−3^ cm s^−1^. The open triangles (Δ) represent the predicted response at 0.1 V s^−1^ but where the currents have been multiplied by a factor of 1.5.

Figure 6A shows a voltammogram for the titanium electrode array in a 1 mM ferrocyanide solution containing 0.1 M H_2_SO_4_ as supporting electrolyte, at different scan rates ranging from 0.01 to 0.1 V s^−1^. Figure 6A shows that a sigmoidal voltammogram is observed at ~+0.5 V. There are two possible origins of this behaviour. First, if the voltammetric timescale is sufficiently short and the rate of heterogeneous electron transfer is fast, then radial diffusion to the individual microcylinders of the array could dominate giving sigmoidal voltammograms. However, for the scan rates used here the depletion layer is significantly smaller (of the order of 0.05 mm) than the separation between adjacent microcylinders (≈0.8 mm) and the depletion zones are not expected to overlap. Second, the rate of heterogeneous electron transfer may be very low, perhaps involving electron tunnelling across the oxide layer (Bewer et al., 1982; Heakal and Awad, 2011), causing the well-defined peaks typically associated with close to ideally reversible couples, such as [Ru(bpy)_3_]^2+^, ferrocene-methanol and ferro/ferricyanide, to become distorted.

Deposition of a gold coating represents a convenient approach to enhancing the rate of heterogeneous electron transfer while preserving the unique geometry that can be achieved using 3D SLM. Moreover, gold deposition creates an electrode surface that is oxide free for potentials less positive than ~+1.2 V vs. Ag/AgCl.

Figure 6B shows scan rate dependent voltammograms obtained for 1 mM ferrocyanide dissolved in 0.1 M H_2_SO_4_ at the gold coated arrays. Despite the fact that HR-SEM indicates that there are negligible differences in geometry of the parent titanium and gold coated electrodes (gold coating is thin which does not significantly increase the thickness of the microcylinders), the response is strikingly different at the gold coated electrodes. Specifically, well-defined peaks are observed, the peak-to-peak separations, ΔE_p_, at low scan rates are <80 mV and the ratio of oxidation to reduction peak currents is one. While larger than the 57 mV expected for an ideal reversible reaction under semi-infinite linear control, the ΔE_p_ value is consistent with a quasi-reversible process. The open circles show the best fit to the 0.01 V s^−1^ experimental data assuming semi-infinite linear diffusion control where there is only one freely adjustable parameter, the standard heterogeneous electron transfer rate constant, k^o^. A satisfactory fit is obtained where k^o^ is 8.0 ± 0.4 × 10^−3^ cm s^−1^. The fact that the response at the gold coated array is consistent with semi-infinite linear diffusion control at the scan rates investigated suggests that the sigmoidal response observed at the *titanium* arrays arises due to a small k^o^. In contrast, when the 0.1 V s^−1^ response is simulated using a k^o^ of 8 × 10^−3^ s^−1^, the peak potentials are satisfactorily predicted, but to match the peak currents, the theoretical response must be multiplied by a factor of 1.5. This result suggests that at the higher scan rate where the depletion layer is thinner, surface roughness significantly influences the voltammogram observed.

#### Co-reactant ECL at Titanium and Gold Coated Arrays

**Figure 7:**
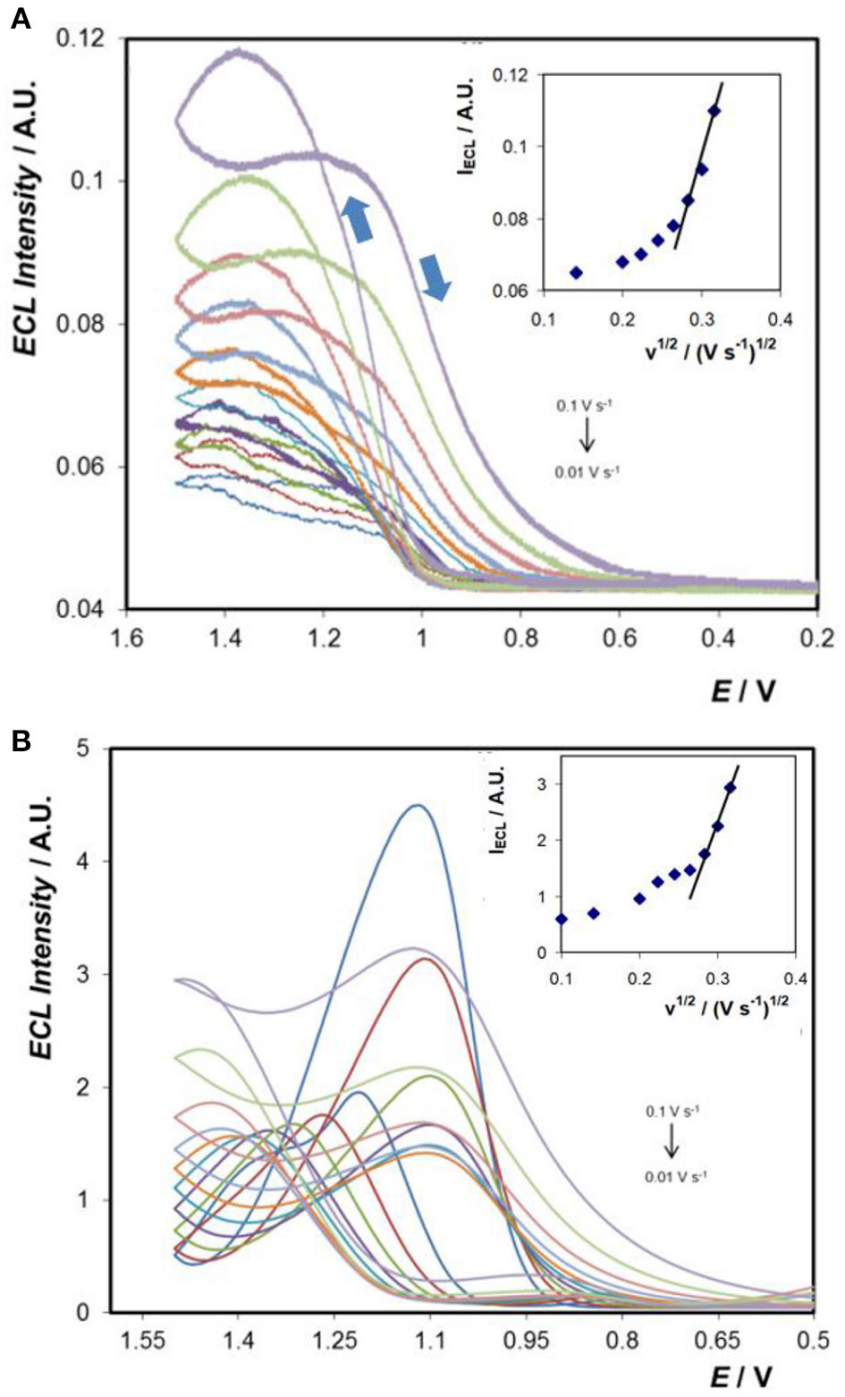
ECL generated at **(A)** 3D Ti electrode and **(B)** Au-coated 3D Ti electrode in 0.1 M PBS (pH 7.4) solution containing 50 μM [Ru(bpy)_3_]^2+^ and 5.0 mM TPA, at scan rates ranging from 0.01 to 0.1 V s^−1^. The insets show the dependence of the maximum ECL intensity on the scan rate for each electrode.

Figures 7A,B shows the ECL responses obtained at the bare- and Au-coated 3D Ti electrodes using TPA as the co-reactant. Significantly, both electrode arrays produce ECL and the intensities increase with increasing scan rate in the range 0.01 to 0.1 V s^−1^ (insets). The onset potential for ECL is ~ +1.1 V for both the bare- and Au-coated 3D Ti electrodes which is consistent with ECL being generated by reaction of Ru^3+^ with the TPA radical.

#### Titanium Array

The electrocatalytic current observed at the titanium array for solutions containing 50 μM [Ru(bpy)_3_]^2+^ and 5.0 mM TPA varies linearly with the square root of the scan rate, ν, at least for 10 < ν <100 mV s^−1^. Significantly, as shown by the inset of Figure 7A, the ECL intensity, I_ECL_, does not show the same behaviour. For 70 < ν <100 mV s^−1^ I_ECL_ increases approximately linearly with increasing square root of the scan rate. Given the very high concentration of TPA:Ru^2+^ used, 5 mM:50 μM, the generation of the co-reactant is not expected to limit the ECL intensity. At slow scan rates, e.g., 10 mV s^−1^, the ECL response changes shape significantly, becomes sigmoidal, exhibits a steady-state response and depends only weakly on the scan rate. These observations are consistent with enhanced mass transport, e.g., of the [Ru(bpy)_3_]^2+^ luminophore and TPA co-reactant above that expected from semi-infinite linear diffusion. A scan rate of ~0.2 mVs^−1^ would be required for the depletion zones established around each of the individual electrodes to coalesce. Thus, it appears that at the slow scan rates, the 3D character of the array influences the ECL intensity. Significantly, in contrast to the current response, this result is more consistent with radial diffusion to the individual microcylinders influencing I_ECL_ due to the multiple kinetic and mass transport processes involved in light, as opposed to current, generation. One notable feature of Figure 7 is the fact that at high scan rates (0.1 V s^−1^) the ECL intensity crosses over and the intensity on the backward scan (from +1.5 V towards 0.2 V) is higher for some potentials than the forward going scan. ECL generation requires *both* Ru^3+^ and the TPA radical to be present simultaneously. Differences in oxidation potentials (Ru^3+^ more positive than TPA), heterogeneous electron transfer rates, stabilities as well as mass transport to the 3D array influence the cross over behaviour. When the scan rate is high, the time constant for transport and reaction of Ru^3+^ and TPA radical is larger than that of the experiment and these reagents remain available to generate light when the potential direction is switched which leads to cross over.

#### Gold Coated Titanium Array

Comparing Figures 7A,B, the ECL intensity increases dramatically, by a factor of over 30 at high scan rate, and by ~100-fold at slow scan rates, for the gold coated titanium array. The significant enhancement is consistent with the large increase in k^o^ for the gold coated array, 8.0 ± 0.4 × 10^−3^ cm s^−1^, compared to 1.1 × 10^−4^ cm s^−1^ for the as printed titanium array. This ~70-fold increase in the rate of heterogeneous electron transfer leads to an increased flux of the luminophore and hence a higher ECL intensity.

Despite the more ideal electrochemical properties of the gold coated array, the ECL peak shape is highly complex and significant hysteresis between the forward and backward scans is observed even at the slowest scan rates. Typically, ECL begins at ~+0.95 V and gives rise to a well-defined peak. On the return scan, the response crosses over the positive going wave before rapidly decreasing to a very low light intensity. As shown in the inset, the dependence of I_ECL_ on the square root of the scan rate is reasonably linear at higher scan rates before becoming significantly less sensitive at the lower scan rates. As discussed for the unmodified titanium array, this behaviour is consistent with the 3D character of the array giving rise to enhanced mass transport at relatively slower scan rates. The generation of electrochemiluminescence is a complex process involving heterogeneous electron transfer, mass transport of both the luminophore and the co-reactant, cross-reaction of the active forms of the co-reactant and the luminophore as well as excited state dynamics. Moreover, any change in one parameter can influence all the other parameters since they are highly co-dependent and interconnected. Modelling the responses obtained here using well-established finite element simulations is especially challenging given the complex geometry that significantly influences the scan rate dependence of the ECL intensity. Recent developments that combine a mechanistic model with a genetic algorithm show significant promise for modelling these complex systems (Rivera et al., 2020).

## Conclusions

This work describes the fabrication and characterisation of a 3D titanium electrode array as well as their electrochemical and electrochemiluminescence properties. Significantly, well-defined microcylinder arrays have been created using selective laser melting where the microcylinder dimensions and inter- microcylinder separations are <800 μm. While these dimensions mean that the internal volume of the array can be exhaustively electrolysed within 150 s, the current response at scan rates between 10 and 500 mV s^−1^ is dominated by semi-infinite linear diffusion to the sides of the cylinders rather than radial diffusion to the tips. Significantly, water electrolysis is inefficient which allows [Ru(bpy)_3_]^1+^ to be generated in aqueous solutions which is not possible using conventional metal electrodes. Also, the sequential generation of [Ru(bpy)_3_]^3+^ and [Ru(bpy)_3_]^1+^ enables electrochemiluminescence to be generated by annihilation avoiding the need to add a co-reactant. This opens up the possibility of developing “reagentless” assays for ECL quenchers including hormones, pharmaceuticals, neurotransmitters, redox active inorganic materials, and drugs of abuse. The rate of heterogeneous electron transfer is slow due to the presence of an oxide layer. However, we demonstrate that the electrodeposition of a thin, conformal, gold layer enhances the rate of electron transfer by more than 80-fold. Significantly, using tri-propyl amine as a co-reactant, the electrochemiluminescence intensity is significantly enhanced by the gold coating. The 3D structure of the array has significant advantages including a large effective surface area while minimising the footprint of the light generating area, enhancing mass transport by shortening the diffusion length between electrodes and allowing the experimental timescale to be used to control the areas of the array that emit light, i.e., only the tips of the microcylinders at short timescales and the complete 3D volume of the array at long times. The ability to tune the interfacial properties of the 3D array after fabrication is highly desirable since the properties can then be optimised for a given application such as sensing, electrocatalysis etc. In particular, rough surfaces such as those developed here that are functionalised with metals such as gold may enable plasmonically enhanced ECL by controlling the radiative and non-radiative decay pathways for the electronically excited state.

## Data Availability Statement

Datasets are available on request. The raw data supporting the conclusions of this article will be made available by the authors, without undue reservation.

## Author Contributions

RF and GW: conceptualisation. SD, SB, MD, LC, GW, and RF: methodology. SD, SB, and MD: investigation. GW, EI, and RF: resources. SD, LC, and RF: data analysis. GW, EI, LC, SD, RF, and ZY: validation and formal analysis. SD, SB, and ZY: visualisation. SD and RF: writing—original draft preparation. SD, MD, LC, GW, EI, and RF: writing—review and editing. GW, RF, EI, and LC: supervision. SD, GW, and RF: funding acquisition. All authors contributed to manuscript revision, read, and approved the submitted version.

## Conflict of Interest

The authors declare that the research was conducted in the absence of any commercial or financial relationships that could be construed as a potential conflict of interest.
