# Supplementary material for: Electrochemiluminescence at 3D Printed Titanium Electrodes
Source: Front Chem. 2021 May 25;9:662810. doi: 10.3389/fchem.2021.662810 (PMC8186460; doi:10.3389/fchem.2021.662810)
Supplement: Supplementary file 1 [file Data_Sheet_1.PDF]

SUPPLEMENTARY INFORMATION

Electrochemiluminescence at 3D Printed Titanium Electrodes

Samantha Douman, Miren Ruiz De Eguilaz, Loanda Cumba, Stephen Beirne, Gordon G. Wallace, Zhilian Yue, Emmanuel I. Iwuoha, and Robert J. Forster

Structural Characterisation. Figure S1A shows backscattered SEM images of the Ti 3D electrodes consisting of dominant brighter regions (most likely Ti) and darker regions perhaps suggesting other materials, such as oxides or binders for the laser based deposition, may be present. EDX was used to probe the elemental composition of the electrodes.[15] Figure S2A shows that the EDX spectrum is dominated by peaks associated with Ti but carbon peaks are also present due to adventitious impurities. Aluminium and vanadium are also observed and are a component of the powder used to create the 3D electrodes.

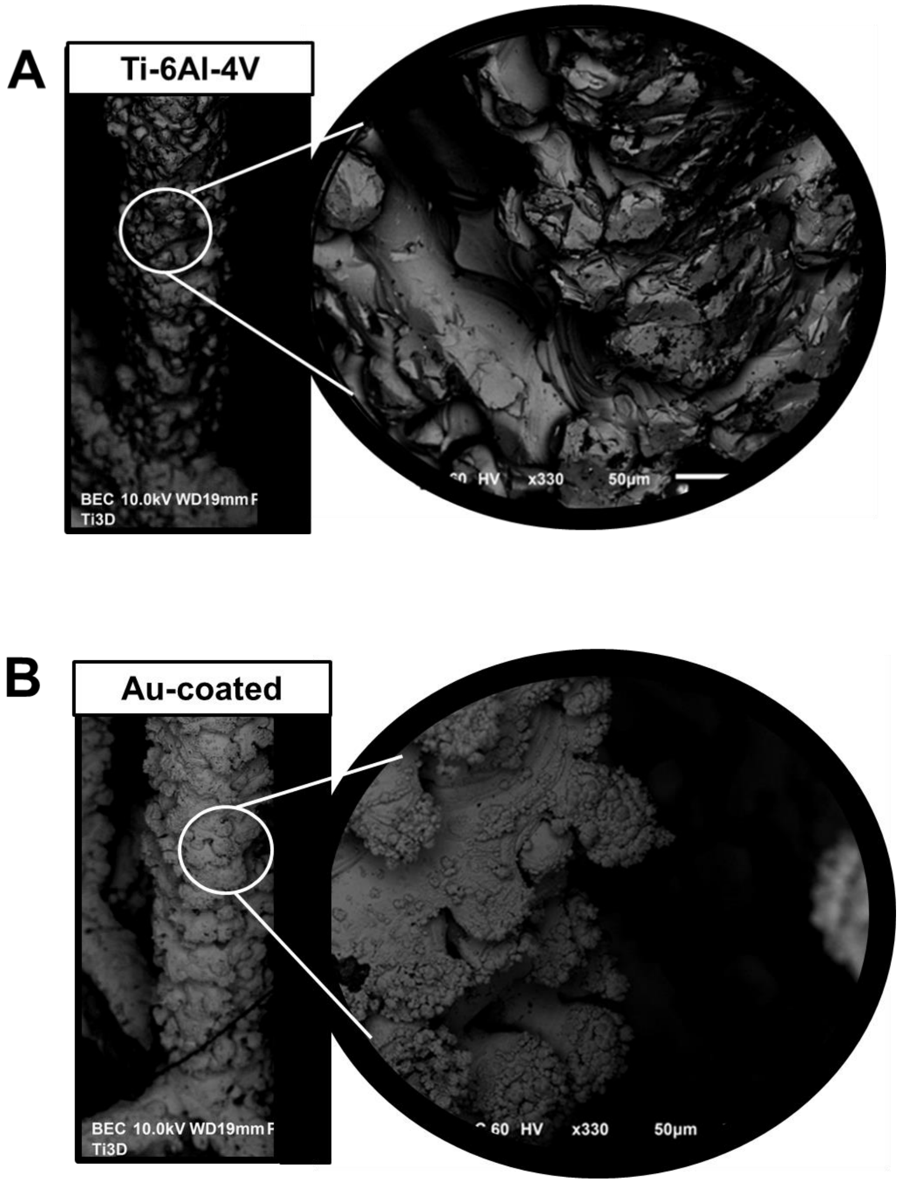

Figure S1. Backscattered SEM images showing topography/surface roughness and the compositional differences (material contrast) between the 3D Ti (A) and Au-coated 3D Ti (B) electrodes, at low and high magnifications using a 10 kV accelerating voltage, respectively. Heavier Au atoms (B) scatter more electrons back towards the detector than the lighter Ti atoms (A) and therefore appear brighter in contrast in SEM image.

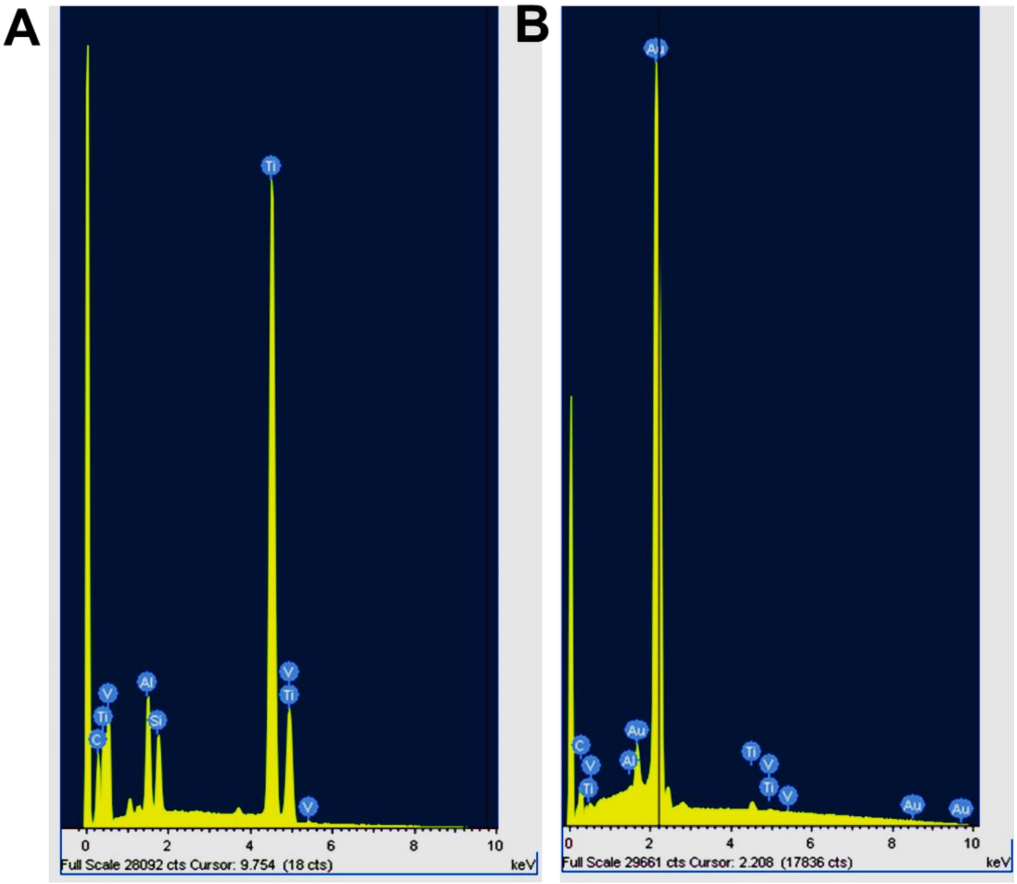

Figure S2. FE-SEM coupled EDX spectrum showing the elemental composition of the 3D Ti electrode (A) and Au-coated 3D Ti electrode (B), respectively.
